# Common marmosets are sensitive to simple dependencies at variable distances in an artificial grammar

**DOI:** 10.1016/j.evolhumbehav.2018.11.006

**Published:** 2019-03

**Authors:** Stephan A. Reber, Vedrana Šlipogor, Jinook Oh, Andrea Ravignani, Marisa Hoeschele, Thomas Bugnyar, W. Tecumseh Fitch

**Affiliations:** a Department of Cognitive Biology, University of Vienna, Althanstrasse 14, 1090 Vienna, Austria; b Department of Philosophy, Lund University, Helgonavägen 3, 22 100 Lund, Sweden; c Artificial Intelligence Lab, Vrije Universiteit Brussel, Pleinlaan 2, 1050 Brussels, Belgium; d Acoustics Research Institute, Austrian Academy of Sciences, Wohllebengasse 12-14, 1040 Vienna, Austria

**Keywords:** Language evolution, Interdependencies, Familiarization-discrimination, Automated video coding, *Simiiformes*, Familiarity preference

## Abstract

Recognizing that two elements within a sequence of variable length depend on each other is a key ability in understanding the structure of language and music. Perception of such interdependencies has previously been documented in chimpanzees in the visual domain and in human infants and common squirrel monkeys with auditory playback experiments, but it remains unclear whether it typifies primates in general. Here, we investigated the ability of common marmosets (*Callithrix jacchus*) to recognize and respond to such dependencies. We tested subjects in a familiarization-discrimination playback experiment using stimuli composed of pure tones that either conformed or did not conform to a grammatical rule. After familiarization to sequences with dependencies, marmosets spontaneously discriminated between sequences containing and lacking dependencies (‘consistent’ and ‘inconsistent’, respectively), independent of stimulus length. Marmosets looked more often to the sound source when hearing sequences consistent with the familiarization stimuli, as previously found in human infants. Crucially, looks were coded automatically by computer software, avoiding human bias. Our results support the hypothesis that the ability to perceive dependencies at variable distances was already present in the common ancestor of all anthropoid primates (*Simiiformes*).

## 1 Introduction

Dependency sensitivity, defined as the ability to perceive that two non-contiguous sensory items are related, i.e. belong to the same conceptual class (Ravignani, Sonnweber, Stobbe, & Fitch, 2013) is important for many aspects of human cognition and considered indispensable in human music and language (Gebhart, Newport, & Aslin, 2009; Newport & Aslin, 2004; van Heugten & Shi, 2010). Human language and music rely on detecting a wide variety of relationships between temporally or spatially non-adjacent elements. In phonology, these relationships could include both close relationships, such as similarity within the syllables in a word (e.g. “vowel harmony”), or more distant relationships across words or even sentences (e.g. rhyme at the end of consecutive phrases). In morphology or syntax, there are an even greater variety of agreement phenomena (e.g. gender agreement in nouns, or verb inflection according to tense) which can effect both neighboring words and arbitrarily distant words (Fitch & Friederici, 2012).

While the presence of all inter-dependent elements is essential for stimulus structure, the distance between them usually is not. Human infants (17 months old) are already capable of detecting non-adjacent dependent elements within a natural stimulus of variable length (van Heugten & Shi, 2010). The evolutionary origin of the cognitive ability to perceive “dependencies at a distance” can be investigated through playback experiments in non-human animals (Fitch, Huber, & Bugnyar, 2010) and, as an important first step, in other non-human primate species.

Previous comparative studies have demonstrated that non-human primates could learn to identify dependencies. Chimpanzees (*Pan troglodytes*) generalized rules in a visual dependency learning task (Sonnweber, Ravignani, & Fitch, 2015) and cotton-top tamarins (*Saguinus oedipus*) learned to recognize visual dependencies (Versace, 2008; Versace, Rogge, Shelton-May, & Ravignani, 2017). In the auditory domain, cotton-top tamarins could recognize dependencies at fixed distances (Newport, Hauser, Spaepen, & Aslin, 2004) and chimpanzees were sensitive to the relation of specific “edge” elements (Endress, Carden, Versace, & Hauser, 2010). In an acoustic familiarization-discrimination experiment, common squirrel monkeys (*Saimiri sciureus*) discriminated between sequences of pure tones which either contained or lacked dependencies (Ravignani et al., 2013), similarly to human infants (van Heugten & Shi, 2010). Notably, because a familiarization-discrimination paradigm was used rather than operant training in the latter study, the subjects' reactions were spontaneous.

Particularly because of this paradigm, Ravignani et al.'s (2013) findings in squirrel monkeys, a New World monkey (*Platyrrhini*), suggest that the ability to recognize dependencies at variable distances may have been present in the common ancestor of all anthropoid primates (*Simiiformes*), the Old and New World monkeys. Despite the apparent importance of dependency sensitivity in human communication, this ability is not necessarily only related to vocal flexibility, but could potentially be linked to other cognitive capacities in primates. However, to date we do not know if squirrel monkeys are the only species in this group to recognize dependencies at variable distances. Thus, in order to evaluate this hypothesis, we conducted a follow-up study with common marmosets (*Callithrix jacchus*), small monkeys belonging to a different family, the Callitrichidae, of New World monkeys (Roos & Zinner, 2016). In the process, we employed a novel rigorous approach to both stimulus design and behavioral analysis.

We used short (~225 ms) sine waves as acoustic elements to create two stimulus classes, which were low pitched (“L”) and high pitched (“H”) pure tones. The stimulus sequences, as in the squirrel monkey study, were patterned according to the grammar LH^n^L, which is a simple finite-state grammar with a dependency between the first and last item and *n* repetitions of elements of another class in between (Ravignani et al., 2013). We employed a ‘familiarization-discrimination’ paradigm: In the ‘familiarization’ phase, we exposed the subjects to sequences of different lengths consistent with the target pattern. The subsequent ‘discrimination’ phase contained two different tests. In one test, the subjects heard i) sequences consistent with the target pattern of familiar and unfamiliar lengths, and ii) sequences inconsistent with the pattern due to missing the initial or final element of the dependency (e.g. H^n^L or LH^n^). In the other test, the positions of the two stimulus classes was reversed, meaning that a sequence HL^n^H was considered consistent and a sequence missing the first or last “H” (i.e., L^n^H, HL^n^) was considered inconsistent (for details see Table 1). We performed the second test to determine, if it turned out that the marmosets had learned a rule about the stimuli, whether they had learned an absolute (only sequences that start and end with “L” are grammatical, i.e., LH^n^L) or relative rule (same-different-same, i.e. AB^n^A) during the familiarization phase.

**Table 1:** Acoustic stimulus sequences used in the familiarization phase, the re-familiarization phase (a), and the two tests in the discrimination phase (b). L signifies a low-pitched tone; H signifies a high-pitched tone; superscript digits signify number of tone repetitions within a stimulus; numbers in brackets signify the number of stimuli with this specific composition in cases where the composition occurred more than once.

a)			
Stimulus class		Stimulus structure	
Familiarization		LHL (160), LH^2^L (120), LH^4^L (80)	
Re-familiarization		LHL (60), LH^2^L (30), LH^4^L (30)	


b)			
Stimulus class	Subclass	Test 1 stimulus structure	Test 2 stimulus structure
Consistent	repetition	LHL, LH^2^L, LH^4^L (2)	HLH, HL^2^H, HL^4^H (2)
	variation	LH^3^L (2), LH^5^L (2)	HL^3^H (2), HL^5^H (2)
Inconsistent	missing first	HL, H^2^L, H^3^L, H^4^L	LH, L^2^H, L^3^H, L^4^H
	missing last	LH, LH^2^, LH^3^, LH^4^	HL, HL^2^, HL^3^, HL^4^

We designed the setup and stimuli in this study with two main aims: i) to improve the original study design used in Ravignani et al. (2013) while keeping it similar enough to make the two experiments comparable, ii) to enable standardization of stimulus exposure, for future comparison studies. In familiarization-discrimination paradigms, the reactions of subjects are spontaneous and not reinforced by training. If using stimuli of low salience, behavioral responses of subjects can be expected to be fairly limited in magnitude. Therefore we ensured that our setup design allowed us to standardize the context of stimulus exposure to minimize behavioral variability. The video recordings of all trials were in fact so similar that custom-made software could automatically code all head turns towards the loudspeaker (Oh et al., 2017).

We hypothesized that general cognitive abilities of anthropoid primates could explain the result in the previous study (Ravignani et al., 2013). We thus predicted that marmosets would be capable of discriminating between sequences with and without dependencies.

## 2 Methods

### 2.1 Subjects and keeping facility

**Fig. 1:**
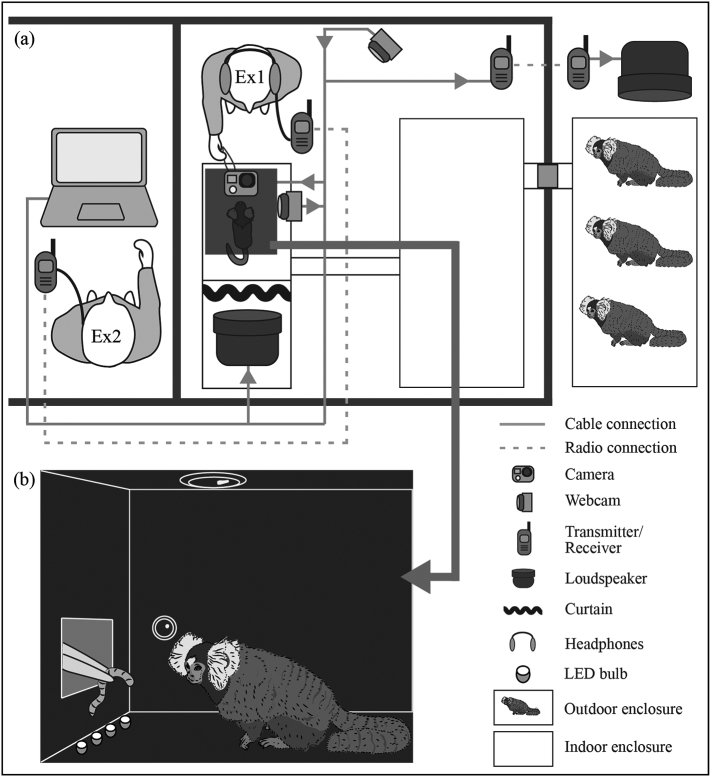
Schematic experimental setup: Two experimenters (Ex1 and Ex2) were in radio contact throughout testing. Ex1 interacted directly with the subject while Ex2 ran the experiment remotely from a different room. During testing, non-subject animals were in the outdoor enclosure without access to the indoor enclosure (a). Subjects were tested in a custom-built experimental box, which was open at its back, placed inside the experimental cage. Ex1 attracted the subject's attention by providing food rewards through a small opening at the front of the box (b). Subjects were filmed from above. Ex2 could observe the animal via the webcam in the side wall of the box. For detailed procedure see main text.

We tested eight adult, captive-born common marmosets (five males) kept in two family groups at the Department of Cognitive Biology at the University of Vienna, Austria. Each group had free access to an indoor and an outdoor enclosure (both 250 × 250 × 250 cm). The indoor enclosure had coniferous wood pellet flooring, and both areas were equipped with a variety of enrichment objects (branches, ropes, platforms, blankets, and sleeping baskets). An experimental cage (150 × 40 × 110 cm) was connected to the indoor enclosure via a runway system with tunnels and sliding doors (Fig. 1a). The two tested groups were housed in different rooms. Each of them shared their room with and had acoustic access to a second non-experimental family group, but a visual barrier hid non-family members from view.

### 2.2 Maintenance and ethics

The animals were fed daily at noon with a varied diet consisting of different fruits, vegetables, pellets, marmoset jelly and gum, protein and vitamin supplements, and a variety of insects. Water was provided ad libitum. The daylight period for artificial lights in the indoor enclosure was maintained on a stable 12-h light/dark cycle. However, during the light cycle (6:30–18:30), the main light source was daylight, which fluctuated according to the natural light/dark cycle of Vienna. Heat lamps were always available. The temperature was maintained at 24–26 °C, and humidity was kept at 40–60%. All individuals were already familiar with their group's experimental cage and with temporary separation from their family from previous studies (e.g. Šlipogor, Gunhold-de Oliveira, Tadić, Massen, & Bugnyar, 2016). During experiments, we lured marmosets to different enclosures exclusively by attracting them with preferred pieces of food. The animals were not food or water deprived at any time, and the session would end at any sign of distress from the subject. The housing conditions and the experimental design were in accordance with Austrian legislation and the European Association of Zoos and Aquaria (EAZA) husbandry guidelines for Callithrichidae*.* The research complied with protocols approved by the institutional board for animal experimentation (Animal Welfare Board Ethical Approval Number 2015–012).

### 2.3 Stimuli

#### 2.3.1 Pure tone frequencies and durations

The audiogram of common marmosets follows the W-shape of other New World primates (Osmanski & Wang, 2011). We selected frequencies for the pure tone stimuli from the two frequency ranges of greater auditory sensitivity centering around 2 kHz and 11 kHz. To the best of our knowledge, empirical data are lacking to date on frequency discrimination in common marmosets, but are available for squirrel monkeys (Wienicke, Hausler, & Jurgens, 2001). Given the similarities between the hearing curve shape of the two species (Heffner, 2004; Osmanski & Wang, 2011), we can predict that marmosets should be capable of perceiving frequency differences of ~70 Hz (2 kHz class) and ~50 Hz (11 kHz class), respectively (Wienicke et al., 2001). In the present study, pairs of randomly synthesized stimulus tones for the 2 kHz class differed on average by ~135 Hz, which would correspond to approximately double the minimally perceivable frequency difference. Forty-four pure sine wave tones for the low frequency class were sampled between 1800 and 2200 Hz (2 kHz ± 10%) and 44 tones for the high frequency class between 9900 and 12,100 Hz (11 kHz ± 10%). The tones of both pitch classes were between 210 and 240 ms long (225 ± 15 ms). The stimuli were synthesized using custom Python scripts (programmed by AR). The time between two pure tone onsets within a sequence was always 250 milliseconds (i.e., because of varying stimulus lengths this meant there were 10–40 ms of silence between tones).

#### 2.3.2 Familiarization phase

The sequences in the familiarization phase were all composed with the rule LH^n^L. A sequence was broadcasted every 5 s. In the study on squirrel monkeys, the number of intervening High tokens, *n*, was either 1, 2, or 3 (Ravignani et al., 2013). In contrast, in the familiarization phase of this experiment, we used 1, 2, or 4 high frequency tones per sequence to prepare for the control playbacks (stimulus subclass ‘variation’, Table 1b) in the discrimination phase. Classically, generalization to a pattern is tested by exposing subjects to longer sequences (higher ‘n’) than the familiarization sequences. Because we used *n* = 3 and *n* = 5 in the present study, the length of novel sequences could be either longer or shorter than the familiar length from the familiarization phase. In total, the familiarization playback contained 360 sequences and lasted 30 min; 160 sequences with *n* = 1, 120 with *n* = 2, and 80 with *n* = 4. The proportion of the stimuli in the familiarization phase ensured that the transitions from H → L (and L → H) occurred as often as from H → H. This measure prevented discrimination by the likelihood of individual transitions rather than the overall pattern. In addition to the full-length familiarization playback track, a shorter ‘re-familiarization’ track was created, which lasted 10 min and was composed in the same manner (Table 1a). For each of the two family groups, a new sample of familiarization sequences was created and all files were concatenated into one playback track in randomized order.

#### 2.3.3 Discrimination phase

The same pure tones from the familiarization phase were used to create the novel stimulus sequences for the discrimination phase. The sequences in Test 1 followed the rule LH^n^L and contained 16 trials: 8 with playbacks consistent, and 8 with playbacks inconsistent with the familiarization pattern. The consistent stimuli included novel sequences of the same length (“repetition”) as in the familiarization phase, and it also contained novel lengths (“variation”) with n set to 3 or 5. The inconsistent stimuli had lengths of *n* set from 1 to 4 and were either missing the first or the last L element (Table 1b). To examine the generality of the acquired pattern, Test 2 contained the same number of trials with the same composition, but all sequences followed the inverse rule HL^n^H (e.g. HLLH was used instead of LHHL). In both Test 1 and Test 2, the 16 test sequences were presented in a randomized order. Each subject received an individual stimulus set, novel sequences assembled from the same pure tones, for both tests to avoid pseudoreplication.

### 2.4 Experimental procedure

#### 2.4.1 Playback setup

The experimental cage was divided into two compartments: the subject compartment and the playback compartment. In the playback compartment, a loudspeaker was installed (JBL Control 2P, frequency response: 80–20,000 Hz), with the membrane pointing towards the subject compartment. A thin black cloth curtain hid the loudspeaker from view (Fig. 1a). In the subject compartment, a custom-built experimental box (L × W × H = 40 × 30 × 40 cm) was placed. One short end sidewall of the box was set against the outer mesh of the experimental cage and had a square opening (5 × 5 cm) 10 cm above ground, while the other short end lacked a wall. The top of the box was equipped with an LED light and a high-speed stand-alone video camera (GoPro hero3) pointing downward. In addition, four infrared LED light bulbs were flush-mounted into the bottom of the box pointing upward (towards the GoPro camera). A webcam installed in one of the long sidewalls at a height of 12 cm allowed for live monitoring (Fig. 1b). The box was coated with a black sealing paint to enable quick cleaning of the surfaces. In addition, the dark color of the floor created a sharp visual contrast to the white ear tufts of the marmosets, aiding the automated video coding procedure (see Section 2.7.1.). An experimenter (“Ex1”, VŠ), who was familiar to all subjects, was present inside the marmoset room during all trials. She was wearing over-ear headphones and listening to music continuously throughout the experiment. This prevented her from hearing the test stimuli and hence from unintentionally cueing the subjects. An in-ear headset worn under the headphones allowed her to communicate (radio connection) with a second experimenter (“Ex2”, SAR) who was operating the experiment-running laptop outside of the marmoset room (Fig. 1a). The webcam in the box and the loudspeaker were both connected to this laptop; GoPro video recording was controlled wirelessly with a remote. Prior to any testing, all subjects were habituated to the experimental set-up, and trained to enter and stay in the experimental box. First, the whole family groups were given access to the subject compartment with food rewards presented by Ex1 inside the box. Later on, individuals entered this compartment alone and sat in the box while receiving food rewards from the square opening facing the outer fence. To familiarize them with sound playing behind their back, Ex2 played back non-experimental sound files during short feeding breaks. These files were voice recordings of the animal keepers who have close contact with the subjects on a daily basis.

#### 2.4.2 Familiarization phase

On the day before the experiment, the non-experimental family group, sharing the room with the experimental family group to be tested, was kept in their outdoor enclosure. Subjects within the family group scheduled for testing the following day were kept in the indoor home enclosure and the other group members were released into the outdoor enclosure. These subjects were exposed to the familiarization track (Table 1a) within the indoor enclosure for 30 min, played back from the MacBook Pro laptop computer connected to the loudspeaker in the playback compartment (facing the indoor enclosure). On the day of the experiment, this procedure was repeated for a re-familiarization of 10 min before beginning the discrimination phase.

#### 2.4.3 Discrimination phase

All marmosets were kept in their family group's outdoor enclosure except the individual being tested. The subject was lured into the experimental cage with a food treat. Ex2 began recording with the GoPro camera and started the experimental software. Executing the program caused the four small infrared LED light bulbs to flash in a given pattern. Infrared light is invisible to primates but could be recorded with the GoPro camera. The pattern created a distinctive ID code connecting each video with a date and session number, and allowed precise synchronization between the experimental log and the video recording. Ex2 loaded the stimuli, previously prepared for the specific individual, into the software and randomized their order. Ex1 attracted the subject to enter the box by offering food rewards, preferably consisting of a banana-honey smoothie filled into a large syringe and provided by placing the nozzle into the square opening of the box through the fence. If the individual started to lose interest in the reward, a variety of other small food items (pieces of fruit, raisins, mealworms, zophobas) were offered via tweezers (Fig. 1b). Ex2 could monitor the subject's behavior via the webcam in the experimental box's sidewall. As soon as the subject was sitting in the box and licking the nozzle of the syringe or eating off the tweezers (facing Ex1), Ex2 informed Ex1 via radio to interrupt the feeding. Ex1 pulled the reward out of reach and Ex2 played a stimulus through the loudspeaker behind the subject. The LED lights flashed once when the stimulus started to play, and when playback had ended. After a break of at least 15 s the animal was again attracted with the reward, in preparation for the next stimulus presentation. This procedure was repeated for all the 16 trials per session. Immediately following these exposures (within the same session), the trials, which could not be completed (e.g. subjects left the box before the stimulus playback ended), were presented to the subject in the same manner once more without a contextual break. After completing all trials, the subject re-joined its family group and the next individual was separated and tested.

### 2.5 Acoustic isolation

We aimed to keep the stimulus exposure of each individual as consistent as possible, and to avoid non-subjects overhearing playbacks. Several provisions were thus implemented to avoid non-subject animals being exposed to the acoustic stimuli prior to their own trials. Playbacks were only presented to individuals in the indoor areas (familiarization trials in indoor enclosures and test trials in experimental cages). Before playback presentation, non-subject animals were directed to the outdoor enclosures and the doorway was sealed with a custom-built plug consisting of wooden boards and noise-cancelling materials. This measure dampened noises between the enclosures; it was however not sufficient to completely mute acoustic contact between individuals. Hence, in addition, a battery-powered loudspeaker (Anchor Explorer Pro-8000, frequency response: 80–16,000 Hz) was placed next to the outdoor enclosure and used to broadcast broadband white noise to the non-subject animals during stimuli presentations (Fig. 1a). During the familiarization phases, the subjects that were to be tested were present in a group in the indoor enclosure and the white noise ran for the entire stimulus playback track. However, during the testing phases, when all other family members (non-subject animals and subjects not currently tested) were in the outdoor enclosure, we limited white noise exposure to occur only during stimulus presentations, because we found during pre-training that individually separated subjects (in the experimental cage) would show signs of distress if they were completely without acoustic contact to their family group. For that purpose, the outside loudspeaker was connected with Ex2's laptop via a radio transmitter-receiver system (Sennheiser EW 112-p G3-A Band, 516–558 MHz). Prior to any stimulus presentation in the testing phase, Ex2 started a 10 s sound file of white noise (fading in for 1 s, fading out for 2 s). Within this time window the stimulus was presented to the subject, and the white noise masked that sound for the other family members in the outdoor enclosure.

### 2.6 Order of testing

Five animals were first tested with Test 1, and four with Test 2 (one of which we lost due to attrition caused by illness between this phase and subsequent phases of the experiment). After a break of two weeks, the entire experiment was repeated (including familiarization phases) and individuals first tested with Test 1 were tested with Test 2, and vice versa. The order of testing within each group was pseudorandomized and differed between the two tests, avoiding any pattern with respect to age, dominance status, or sex.

### 2.7 Data analysis

#### 2.7.1 Video coding

**Fig. 2:**
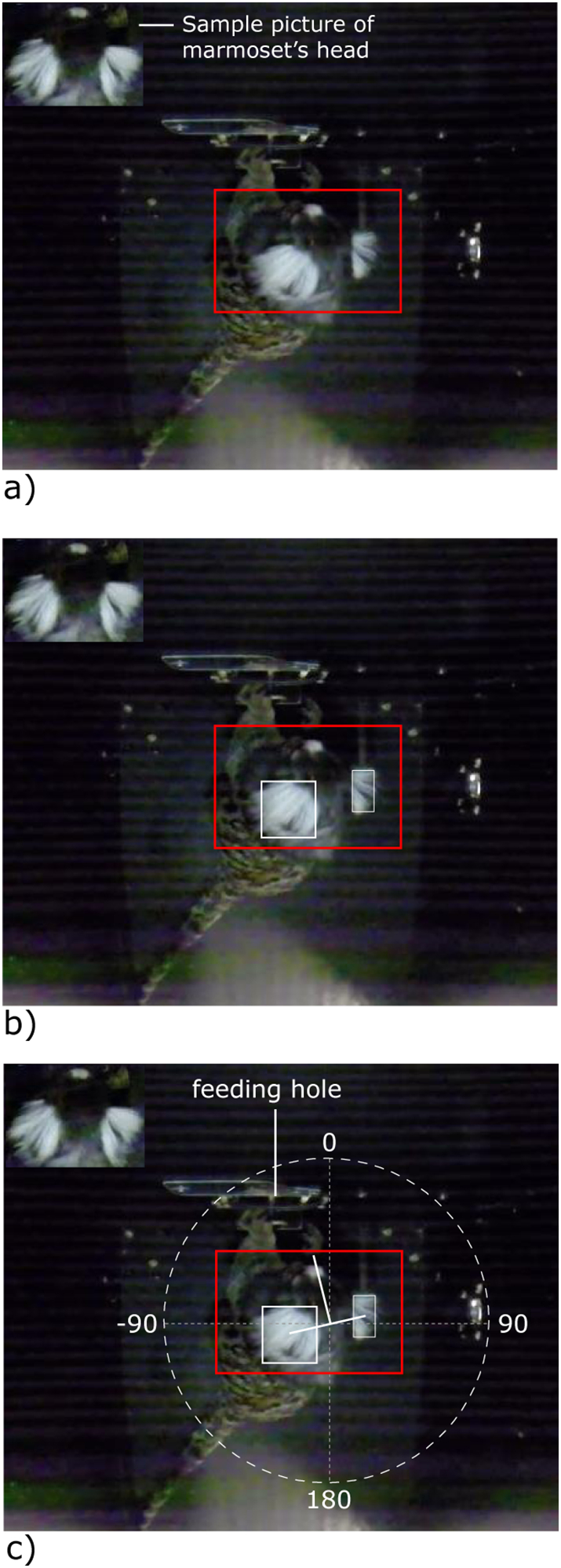
The three steps of obtaining the head direction: a) detecting the head position using SURF algorithm with a sample picture, b) detecting positions of ears using color detection, c) calculating the head direction. When the head direction was at 0° the subject was facing the feeding hole, when the head direction was at 180° the subject was facing the loudspeaker. Crossing the ±90° thresholds was recorded as a full head turn.

The GoPro camera videos of the subject during the experiment, shot from above, were used for behavioral coding. Custom programmed software (Oh et al., 2017) recorded head turns of the subjects for all trials fully automatically. First, the program identified the head of the marmoset with the “SURF” algorithm (Oh et al., 2017) using a template image (photograph of a marmoset's head from above). Because the animal could enter the box only from one side, the program also gained and stored the information on the subject's initial looking direction. As the surface of the experimental box had been painted black, the white ear tufts of the marmosets stood in strong visual contrast to the dark background. Hence, after the program had found the head's position in the video, it used color detection in this general area to locate the ear tufts. The software then extrapolated the current looking direction from the ear position, constrained by initial looking direction (Fig. 2). In an output file produced to manually check the results of the algorithm, the program indicated the presumed gaze direction by displaying a line running from the centre of the subject's head towards the calculated point of visual attention (see Supplementary Video S1). For every frame of the video (100 frames/s) the program recorded the angle of the head direction in relation to the front wall of the box (feeding opening): facing the front directly was defined as 0°; the loudspeaker was located at 180°, and consequently a head turn (± 90°) was coded when the head direction line crossed 90° or − 90° starting from facing the front wall in the 5 s following stimulus onset. We also calculated the average and maximum absolute angle. Two authors (SAR & VŠ) went through all 256 videos to evaluate whether the program had made any errors (see Supplementary Video S1 for an example, note that in the video the mathematical classification of the angles was used, a 90° angle corresponds to straight to the front). In a few instances the head direction line had to be corrected by switching it by 180°, as the program had identified the ear tufts correctly but erroneously calculated the back of the head as being the front. On occasion, the program misrepresented the actual line of head direction, as the animal had tilted the head sideways making one ear tuft invisible to the camera. In such cases the angle value of the last frame with correct assessment was used and maintained until the program could again use the visual information for both tufts. These corrections were very rare (<1% of all frames) and the pure data output of the software and the manually corrected version differed only by one single head turn by one individual (Oh et al., 2017).

#### 2.7.2 Statistical analysis

For both tests (Test 1 & Test 2), the ‘mean head orientation’ (average angle in degrees), the ‘maximum head rotation’ (maximum angle in degrees), and the total ‘number of head turns’ per stimulus subclass (repetition, variation, missing first, missing last), occurring after the stimulus had finished playing, were calculated. For each head turn, the latency was recorded as well. These four measurements (‘mean head orientation’, ‘maximum head rotation’, ‘number of head turns’, ‘latency until head turning’) were used as response variables in Generalized Linear Mixed Models (GLMMs) with subject identity nested within group identity as a random factor. The four stimulus categories were assigned to two levels of the factor ‘consistency’ with the grammar of familiarization: ‘repetition’ and ‘variation’ being ‘consistent’ test stimuli, and ‘missing first’ and ‘missing last’ being ‘inconsistent’ with the familiarization pattern. The ‘subject order’ within a testing day served as an additional factor. The models looked at the influence of ‘test type’ (Test 1/Test 2), ‘consistency’ (consistent/inconsistent), ‘subject order’ (1–3), the interaction between ‘subject order’ and ‘consistency’, and ‘test type’ and ‘consistency’.

Additional GLMMs investigated whether the number of repeats of the non-dependent elements in the stimulus subclass ‘variation’ (3 or 5, see Table 1b) had an influence on the head turning behavior. In these models, the factors were ‘test type’ (Test 1/Test 2), ‘number of repeats’ (3/5), and their interaction term.

The best models were chosen using the Akaike Information Criterion (AIC). Post-hoc analyses were conducted using single-term GLMMs. Exact Wilcoxon signed-rank tests (Mundry & Fischer, 1998) were used for within-subject comparisons, for which effect sizes (Cohen, 1988) were calculated. All analyses were performed in R (version 3.0.2) using the packages ‘lme4’ and ‘coin’.

## 3 Results

**Fig. 3:**
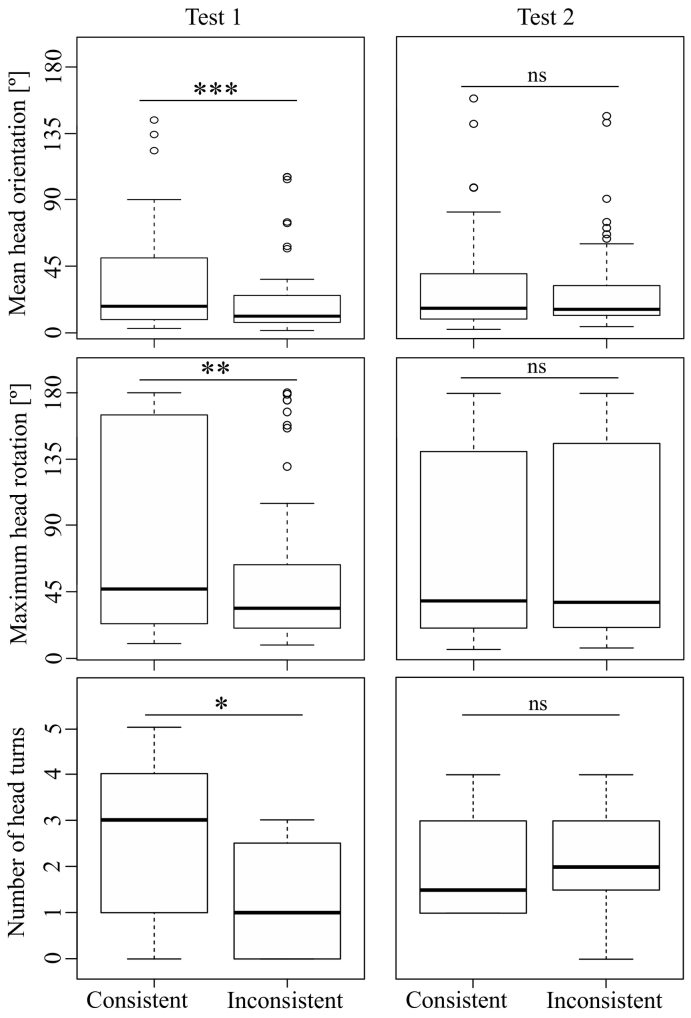
Subjects turned more towards the loudspeaker if they heard stimuli consistent with the familiarization stimuli in Test 1, but not in Test 2. ‘Repetition’ and ‘variation’ represent test stimuli consistent with the familiarization pattern, while ‘missing first’ and ‘missing last’ represent inconsistent stimuli. Boxplots represent 25th and 75th percentiles, the centre line indicates the median, whiskers represent the non-outlier range, and dots are outliers (*P ≤ .05, **P ≤ .01, ***P ≤ .001, ns = non-significant).

The final models for ‘mean head orientation’, ‘maximum head rotation’, and ‘number of head turns’ all contained the fixed effects ‘consistency’ and ‘test type’, and the interaction between the two. Of these, only ‘consistency’ (consistent/inconsistent) always had a significant effect on the response variable (see Table 2). ‘Test type’ (Test 1/Test 2) never had a significant effect by itself while its interaction term with ‘consistency’ resulted in either significance or at least a non-significant trend (see Table 2). Marmosets turned further and more often towards the loudspeaker if they heard stimuli that were consistent with the familiarization pattern than to those that were inconsistent. That is, they showed a looking preference for stimuli following the familiarization pattern. An individual analysis of the two test types revealed that the subjects turned further and more often towards the consistent stimuli in Test 1 (Table 2, Fig. 3); a difference between ‘variation’ and ‘repetition’ was not observed for any orientation variable (exact Wilcoxon signed-rank test, *N* = 8, *z* ≤ −0.842, *P* ≥ .461, *d* ≤ 0.227). However, there was no differential response with respect to ‘consistency’ in Test 2 (Table 2, Fig. 3). The final model looking at ‘latency until head turning’ contained no predictor except for the random factor (transformation = +1; distribution = Gaussian + “log” link).

**Table 2:** Values of the final Generalized Linear Mixed Models for ‘mean head orientation’, ‘maximum head rotation’, and ‘number of head turns’.

Response variable	Data	Distribution	Coefficient	Estimate	SE	*z*	*P*
Mean head orientation	Test 1 & Test 2	Gamma	Test type	0.006	0.005	1.274	0.203
			Consistency	0.017	0.006	2.823	0.005
			Test type*Consistency	−0.015	0.008	−1.808	0.071
Mean head orientation	Test 1	Gamma	Consistency	0.017	0.003	5.506	<0.001
Mean head orientation	Test 2	Gamma	Consistency	0.002	0.005	0.362	0.717
Maximum head rotation	Test 1 & Test 2	Gamma	Test type	0.002	0.002	0.855	0.392
			Consistency	0.007	0.002	2.993	0.003
			Test type*Consistency	−0.007	0.003	−2.203	0.028
Maximum head rotation	Test 1	Gamma	Consistency	0.007	0.002	3.112	0.002
Maximum head rotation	Test 2	Gamma	Consistency	<0.001	0.002	0.106	0.916
Number of head turns	Test 1 & Test 2	Binomial	Test type	−0.39	0.396	−0.984	0.325
			Consistency	−0.988	0.439	−2.251	0.024
			Test type*Consistency	1.151	0.597	1.929	0.054
Number of head turns	Test 1	Binomial	Consistency	−0.971	0.437	−2.224	0.026
Number of head turns	Test 2	Binomial	Consistency	0.165	0.406	0.407	0.684

The ‘number of repeats’ in the control playbacks (3 or 5 central elements) did not affect the subjects' responses. The final GLMMs looking at the stimulus subclass ‘variation’ either solely contained the fixed effect ‘test type’, which had no significant influence (‘mean head orientation’: Estimate = 0.012, SE = 0.007, *z* = 1.699, *P* = .089, distribution = Gamma / ‘number of head turns’: Estimate = −0.126, SE = 0.290, *z* = −0.435, *P* = .663, distribution = Binomial), or only the random factor (‘maximum head rotation’, distribution = Gamma).

## 4 Discussion

Our findings demonstrate that marmosets are sensitive to dependencies at variable distances, much like squirrel monkeys in a previous playback experiment (Ravignani et al., 2013). Using a comparable but improved methodology to the squirrel monkey study, the common marmosets tested here were able to recognize a pattern with dependencies at variable distances by discriminating between sequences matching and violating this pattern. The sequence pattern we used had dependent first and last elements that belonged to one category, while the middle elements belonged to a second category. The number of repetitions of the second category between the two dependent elements from the first category did not influence successful discrimination. Because marmosets were familiarized to only some of the possible sequence lengths, this result is consistent with a generalization of the rule acquired during the familiarization phase. Combined with the data from humans and chimpanzees, these results lend support to the hypothesis that the ability to perceive dependencies at a distance might have already been present in the common ancestor of all anthropoid primates (*Simiiformes*). Generalizing this result to all primates would require testing with prosimians (e.g. lemurs).

An intriguing difference to the squirrel monkeys study is that marmosets reacted with more head turns towards the loudspeaker when they heard stimuli consistent with the familiarization rule, whereas primates often show more head turns in response to violations of the original pattern in playback experiments (Saffran et al., 2008; Wilson et al., 2013). At first glance this is counterintuitive, as the familiarization-discrimination paradigm shares many similarities with ‘expectancy violation’ experiments. In such setups, one usually predicts that unexpected stimuli would cause stronger responses than expected ones (Lewis & Goldberg, 1969). However, a preference for familiarity over novelty has been documented in comparable studies on human infants (Houston-Price & Nakai, 2004). In cross-modal studies, infants typically show a familiarity/consistency preference (Kuhl & Meltzoff, 1982; Meltzoff & Borton, 1979). If the exposure to the familiarization stimuli is brief, subjects may develop a familiarity preference at first (Hunter & Ames, 1988; Wagner & Sakovits, 1986). Continued exposure to the template rule (i.e. familiarization stimuli) can then eventually lead to a novelty preference (Pascalis & de Haan, 2003). Our subjects were exposed to the familiarization stimuli for a maximum total of 80 min (40 min per test, 2 weeks break until second exposure) before Test 1 while being allowed to freely interact with other group members. However, even for human infants it is difficult to predict how much familiarization will be needed to observe discrimination in the testing phase. Overexposure can easily lead to saturation, which can result in the absence of any response to familiar elements, independent of their patterning. In nonhuman primates, consistency preferences during playback experiments have been documented on rare occasions. For instance, rhesus macaques (*Macaca mulatta*) were exposed to videos of conspecific facial expressions during vocalization that either matched or did not match simultaneous playbacks of conspecific vocalizations. In this bimodal experiment, subjects preferred to look at the screen in which the two modalities matched (Ghazanfar & Logothetis, 2003). For the present study, the direction of reaction (i.e. whether the subjects react to consistent or inconsistent stimuli) was not of any concern for the main hypothesis of discrimination between the two stimuli types we were testing. Thus, the observed responses demonstrate the common marmosets' ability to discriminate between acoustic sequences with and without dependencies.

Our subjects did not discriminate between the stimuli classes in Test 2. In this part of the experiment we explored whether the marmosets would react to the lack or presence of dependencies even if the stimulus elements that represented the dependency were reversed compared to the familiarization. While there was tentative evidence that squirrel monkeys in the previous study showed a response in Test 2, this result was weak, and required exclusion of data (Ghirlanda, 2017; Ravignani et al., 2013). However, absence of a differential response does not exclude the possibility of the presence of such a capacity in marmosets, especially as chimpanzees also reached the level of generalization of Test 2 in the visual domain (Sonnweber et al., 2015).

It is interesting to note that the overall shape of our familiarization stimuli followed a “low-high-low” contour reminiscent of many primate vocalizations, and in particular the primate “isolation call” of which the marmoset *phee* call is one example (Newman, 1992). It is thus plausible that marmosets have some predisposition to attend to sequences with this pitch contour and not others, and that this may have an effect on their looking behavior to other contours, a hypothesis which could be tested experimentally. However, we do not think it likely that our animals reacted to the strings of sine tones in our experiment as vocalizations, because they never showed vocal responses to our playback stimuli as they do to vocal playbacks (Norcross, Newman, & Fitch, 1994).

It has been argued that studies on non-human animal pattern perception would profit from using conspecific vocalizations as elements (Gentner, Fenn, Margoliash, & Nusbaum, 2006), because they would be more salient to the subjects. Indeed, common ravens (*Corvus corax*) spontaneously perceive structural changes in sequences of territorial raven calls (Reber et al., 2016). However, such a study design is much more feasible in songbirds than in primates. While songbirds usually have several vocal elements relating to the same behavioral context, e.g. common ravens have at least 79 territorial calls (Enggist-Dueblin & Pfister, 2002), primates tend to have few or only one call type per context. Experiments of pattern perception may be difficult to interpret with conspecific vocalization in non-human primates, because one call type might have a higher salience than another one, leading to a call-dependent reaction. Thus, we recommend using non-conspecific acoustic elements, such as pure tones, for studies on non-human primate pattern perception (Kikuchi et al., 2017; Ravignani & Sonnweber, 2017; Wilson, Marslen-Wilson, & Petkov, 2017). Our study design could have compensated for the reduced salience of the pure tone stimuli because of the detailed automatic coding procedure. For this specific experiment, the responses of the subjects were strong enough for us to focus on full head turns towards the loudspeaker. However, the analysis software is able to extract the head's angle to the loudspeaker for every individual video frame allowing researchers to investigate even very minor responses to the stimuli. Furthermore, the automatic video analysis avoided not only any coding bias, it was also approximately nine times faster than equivalent manual coding (Oh et al., 2017).

In addition to the technological advances of our study, we attempted to improve other aspects of the experiment. Among other things, we made sure that the tested subject had acoustic contact with its group members throughout the discrimination phase with the exception of the precise moments of the stimulus presentations. This resulted in marmosets being calm throughout the sessions. Farther, we avoided involuntary cueing by ensuring that the experimenter interacting with the subjects could not hear the stimuli and that the experimenter playing the stimuli was unaware of which stimulus was about to be played. Finally, we improved the stimulus design by using sequences of intermediate length in the familiarization phase, which allows stronger conclusions to be drawn about generalization than the classic procedure of only using longer stimuli in the testing phase.

In conclusion, we employed a highly controlled setup to replicate and extend a previous study on dependencies at variable distances, conducted with squirrel monkeys, in the common marmoset. We found that common marmosets, like squirrel monkeys (Ravignani et al., 2013), cotton-top tamarins (Versace, 2008; Versace et al., 2017), and chimpanzees (Ravignani & Sonnweber, 2017; Sonnweber et al., 2015) are able to discriminate between sequences with and without dependencies. Our findings suggest that this capacity does not require specific adaptations such as a higher degree of encephalization, and might have been present in the common ancestor of Old and New World monkeys. Further research is needed to identify whether this capacity is also present in other non-human animal species.

The following are the supplementary data related to this article.

Supplementary Video S1 The video shows the window of the revision software running through a recording of a complete trial with a head turn. Supplementary Video S1

Supplementary Material 1 Image 1

Supplementary Material 2 Image 2

## Data availability

Original data are stored on the server of the Department of Cognitive Biology at the University of Vienna. All videos are accessible at https://doi.org/10.5281/zenodo.1464862. The data used for statistical analyses are in the supplementary appendix (‘GLMM_data.csv’).

## Competing interests

The authors declare that they have no competing interests.

## Author contributions

AR and WTF conceived the study, SAR, VŠ, JO, HM, TB and WTF designed it. SAR created the stimuli and analysed the data. VŠ coordinated testing and habituated the animals. JO designed and built the experimental setup, and created the coding software. SAR, VŠ, JO, and MH conducted the experiments. SAR and VŠ wrote the manuscript. AR, MH, TB and WTF co-wrote the manuscript. SAR and JO created the figures. All authors gave final approval for publication.
