# Supplementary material for: Common marmosets are sensitive to simple dependencies at variable distances in an artificial grammar
Source: Evol Hum Behav. 2019 Mar;40(2):214–21. doi: 10.1016/j.evolhumbehav.2018.11.006 (PMC6472617; doi:10.1016/j.evolhumbehav.2018.11.006)
Supplement: Supplementary file 2 — Supplementary Material 1 [file mmc2.pdf]

Code sheet explaining the variables

‘date’: Date of the conduction of the trial (YYYYMMDD)

‘group’: The marmoset group the subject belongs to (Group 1 or 2, “G1” or “G2”)

‘subject’: The individual identity of the subject (“Name”)

‘grammar’: The stimulus sequence of the trial (low-pitched tone “L”, high-pitched tone “H”)

‘test_type’: If the trial was part of “test1”, consistent sequences followed the rule LH^nL; if the trial was part of “test2”, consistent sequences followed the rule HL^nH

‘consistency’: Sequences following the rules LH^nL and HL^nH are “consistent”, sequences lacking the beginning or ending element are “inconsistent”

‘number_of_repeats’: Consistent sequences with an ‘n’ of center elements which did not occur in the habituation phase (‘n’=3 or 5)

‘subject_order’: The order in which the animals were tested on the same day

‘mean_head_orientation’: The average angle over all frames after the stimulus finished playing

‘maximum_head_rotation’: The maximum angle over all frames after the stimulus finished playing

‘number_of_head_turns’: The number of times the head orientation crossed the |90°| threshold after the stimulus finished playing

‘latency_until_head_turning’: The time from the end of the stimulus until the occurrence of a head turn
